# 

**Published:** 1903-03-28

**Authors:** 


					the hospital. "The standard of highest purity."?Lancet. mabch 28th, 1903.
CADBURY'S w*. o to* CADBURY'S
COCOA *\t JIFfl COCOA
ABSOLUTELY PURE. <a*^5Z> MO druos or alkalies used.
No. 861. Vol. XXXIII. L? NewapaperlJ SATURDAY,
March 28th, 1903.
[SubsmptionTenns,] pRI0H TWOPENCE.

				

